# RNA-Seq analysis reveals new evidence for inflammation-related changes in aged kidney

**DOI:** 10.18632/oncotarget.9152

**Published:** 2016-05-03

**Authors:** Daeui Park, Byoung-Chul Kim, Chul-Hong Kim, Yeon Ja Choi, Hyoung Oh Jeong, Mi Eun Kim, Jun Sik Lee, Min Hi Park, Ki Wung Chung, Dae Hyun Kim, Jaewon Lee, Dong-Soon Im, Seokjoo Yoon, Sunghoon Lee, Byung Pal Yu, Jong Bhak, Hae Young Chung

**Affiliations:** ^1^ Molecular Inflammation Research Center for Aging Intervention, Pusan National University, Busan, Korea; ^2^ Department of Predictive Toxicology, Korea Institute of Toxicology, Daejeon, Korea; ^3^ Human and Environmental Toxicology, School of Engineering, University of Science and Technology, Daejeon, Korea; ^4^ GenomicTree Inc., Yuseong-gu, Daejeon, Korea; ^5^ Department of Biology, College of Natural Sciences, Chosun University, Gwangju, Korea; ^6^ Personal Genomics Institute, Genome Research Foundation, Suwon, Korea; ^7^ Department of Physiology, University of Texas Health Science Center at San Antonio, San Antonio, TX, USA

**Keywords:** aging, inflammation, RNA-Seq, differentially expressed genes, novel genes, alternative splicing, Gerotarget

## Abstract

Age-related dysregulated inflammation plays an essential role as a major risk factor underlying the pathophysiological aging process. To better understand how inflammatory processes are related to aging at the molecular level, we sequenced the transcriptome of young and aged rat kidney using RNA-Seq to detect known genes, novel genes, and alternative splicing events that are differentially expressed. By comparing young (6 months of age) and old (25 months of age) rats, we detected 722 up-regulated genes and 111 down-regulated genes. In the aged rats, we found 32 novel genes and 107 alternatively spliced genes. Notably, 6.6% of the up-regulated genes were related to inflammation (*P* < 2.2 × 10^−16^, Fisher exact t-test); 15.6% were novel genes with functional protein domains (*P* = 1.4 × 10^−5^); and 6.5% were genes showing alternative splicing events (*P* = 3.3 × 10^−4^). Based on the results of pathway analysis, we detected the involvement of inflammation-related pathways such as cytokines (*P* = 4.4 × 10^−16^), which were found up-regulated in the aged rats. Furthermore, an up-regulated inflammatory gene analysis identified the involvement of transcription factors, such as STAT4, EGR1, and FOSL1, which regulate cancer as well as inflammation in aging processes. Thus, RNA changes in these pathways support their involvement in the pro-inflammatory status during aging. We propose that whole RNA-Seq is a useful tool to identify novel genes and alternative splicing events by documenting broadly implicated inflammation-related genes involved in aging processes.

## INTRODUCTION

It has been established that chronic inflammation plays a major role in oxidative stress-induced aging and aging-related diseases [1]. Inflammatory processes activated by oxidation are known to produce reactive oxygen species (ROS) that inflict not only oxidative damage, but also elicit the release of additional omnipotent cytokines and chemokines, perpetuating the cycle and resulting in a chronic inflammatory condition during aging, as predicted by molecular inflammation [2, 3] and inflammaging [4, 5]. Chronic inflammation can also result from a disrupted redox balance and weakened anti-oxidative defense system, leading to the activation of redox-sensitive pro-inflammatory transcription factors, such as *NF-κB*, during aging [6]. Thus, the aging process is exacerbated by the up-regulation of interleukin (IL)-1β, tumor necrosis factor (TNF)-α, cyclooxygenase (*COX*), and inducible nitric oxide synthase (*iNOS*), resulting in an organism's vulnerability to an age-related inflammatory status [7]. Although previous studies have revealed possible links between inflammation and aging, there have been no genomic scale evidence for the interaction between specific inflammation pathways and the aging process.

A number of cDNA microarray studies have been carried out in mammals, including mice, rats, and humans, to gain an understanding of the transcriptome during the aging process [8, 9]. Microarrays are powerful tools for analyzing gene expression and have increased our understanding of the intricate biology involved in normal and diseased organisms. In addition, cDNA microarray technology has been used to identify age-related changes in key pathways, such as inflammatory and mitochondrial processes [8–10]. However, they have inherent limitations, including the lack of sensitivity to low abundance transcripts and difficulty in detecting alternative splicing variants and novel transcripts [11, 12]. The capability to detect low abundance transcripts is important, since most gene transcripts are present in low quantities [13, 14]. It is also important to identify novel RNAs, including non-coding RNAs [15, 16].

Here, we utilized advanced RNA sequencing (RNA-Seq) based on next-generation sequencing technology [17]. Using RNA-Seq, we examined age-related differential gene expression of the rat kidney genome. Two experimental groups (six rats in each group) were compared for gene expression levels in aged rats. In addition, novel genes and alternative splicing of mRNAs in aged rats were investigated. We then analyzed gene sets such as differentially expressed genes. We identified the changes and effects of genes related to inflammation in global gene transcription, as well as novel genes and alternative splicing events during the aging process.

## RESULTS

### Detection of altered gene expression and inflammation genes in young and aged rats

**Figure 1:**
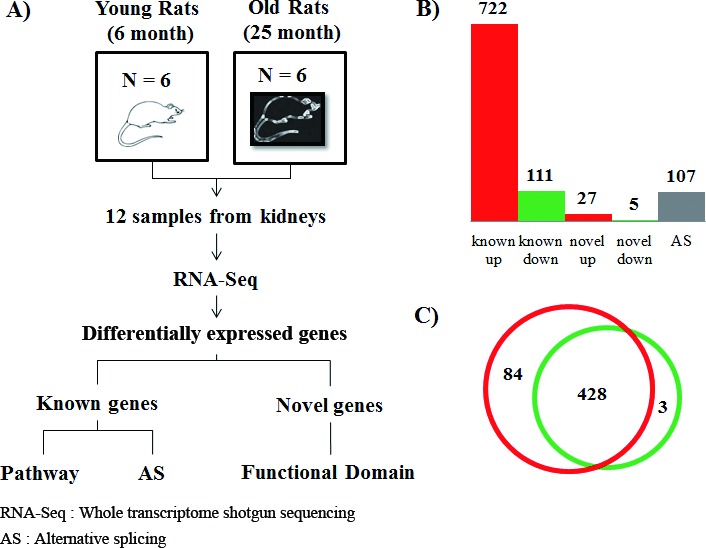
Experimental design and differentially expressed genes with aging **A.** To investigate the molecular basis of aging at the genomic level, we utilized RNA-Seq technology based on Illumina Hiseq-2000. We generated 7.3 billion 99-bp paired-end reads from 12 rats (six young AL, six aged AL). A total of 73% of all reads were successfully mapped on the rat reference genome of NCBI build 5 using both TopHat and Cufflink programs. **B.** Based on the expression levels of known genes, we identified 722 up-regulated and 111 down-regulated genes that were changed by more than 2-fold (FDR < 0.05) in known genes. We carefully analyzed sequences of novel genes using NCBI NT and the domain database after removing non-coding RNA, and found that out of the 322 novel candidates with homologous genes in the NT database, only 32 novel genes had functional domains. The novel genes changed differentially by more than 2-fold. Using the Cufflink program, we selected 108 genes with alternative splicing events that had increased by more than 2-fold by comparing transcripts of young and aged rats. Interestingly, 48 known genes (6.6%), 5 novel genes (15.6%), and 7 genes (6.5%) with alternative splicing events were related to inflammation, showing statistically significant scores (*P* < 0.001, Fisher's exact test). Genes related to inflammation were assigned to ‘inflammation response’ of gene ontology. **C.** To identify regulatory molecules of up-regulated genes (Table 1), we analyzed transcription factors using the TRANSFAC database. We found 84 transcription factors that existed in transcriptional start site (TSS) of up-regulated genes, except transcription factors of down-regulated genes among the 428 transcription factors. In contrast, 3 transcription factors existed only in the TSS of down-regulated genes. The red circle represents transcription factors that existed in the transcriptional start site of up-regulated genes. The green circle represents transcription factors of down-regulated genes. Detailed information is described in Table S4.

We generated 7.3 billion 99-bp paired-end reads from 12 rats (six young Ad libitum (AL), six aged AL) (Figure 1A). Using readings from both TopHat [18] and cufflink [19], 73% of the total RNA reads were successfully mapped to the rat reference genome (NCBI build 5). By comparing against the current NCBI genome annotation, 31,506 known transcripts, spanning a total of 74 Mb, were identified. More than 0.35% of the genes did not match to the rat genome. Based on the expression levels of known genes, we identified 722 up- and 111 down-regulated genes that were changed by 2-fold or more (false discovery rate (FDR) < 0.05) (Figure 1B and Table S2).

To examine the effect of inflammation on aging in terms of RNA expression, we tested the statistical significance of genes related to inflammation in the up-regulated genes in the old rats. 48 genes were found to be statistically significantly related to inflammation (6.6%) (*P* < 2.2 × 10^−16^, Fisher's exact test). Genes related to inflammation were assigned to the ‘inflammation response’ of gene ontology (GO).

### Identification of pathways involving differentially expressed genes

To classify genes in aged transcriptomes, we analyzed predefined biological pathways involving genes that significantly differed between aged and young rats in the gene set enrichment analysis [20]. Representative terms for biological pathways were used in the context of the Kyoto Encyclopedia of Genes and Genomes (KEGG) (http://www.genome.ad.jp). In KEGG terminologies, 20 pathways involved up-regulated genes, while 11 pathways involved down-regulated genes under the filtering option with a *P* < 0.01 and FDR < 0.05 (Table S3).

**Figure 2:**
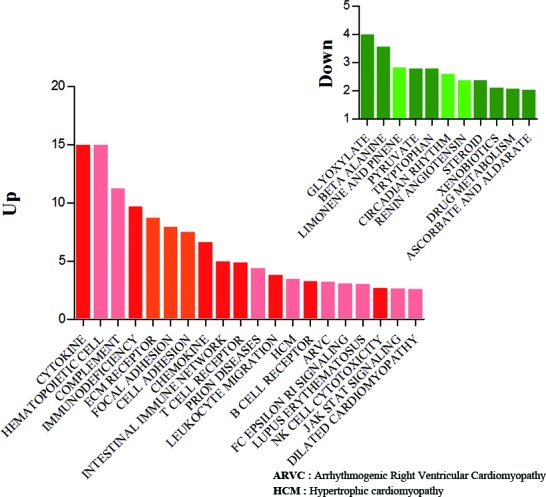
Gene set enrichment analysis of up- and down-regulated genes Classification of the up- (722) and down- (111) regulated genes during aging in the context of KEGG terminologies. The mapping terminologies (pathways) were selected by Fisher's exact *t*-test and false discovery rate (filtering options: *P* < 0.01 and FDR < 0.05). Red bars indicate up-regulated gene sets and green bars represent down-regulated gene sets in each condition. Y bar indicates the modified significant value that was calculated based on the following equation: abs(−log(p-value)). Each graph indicates the distribution of differentially changed genes with respect to the biological process.

In the aged rats, most of the up-regulated genes were involved in inflammation-related pathways, including cytokine and cytokine-receptor interaction, primary immunodeficiency, chemokine signaling, and T-cell and B-cell receptor signaling. In contrast, genes involved in various metabolism and biosynthesis pathways showed marked down-regulation, including glyoxylate and dicarboxylate metabolism, pyruvate metabolism, steroid biosynthesis, and drug metabolism (Figure 2). Because the gene set enrichment test depends on gene sets, we evaluated various pathway terms using BioCarta and Reactome as well as the KEGG pathway. In other databases, most genes involved in energy metabolism show decrease, while most of the inflammation-related pathway genes increase with age.

### Transcription factors involved in the regulation of both inflammation and cancer

To identify regulatory molecules of up-regulated genes using the gene set enrichment test (Table 1), we conducted transcription factor analysis using the TRANSFAC database [21]. We found that 84 transcription factors were present at the transcriptional start site (TSS) of up-regulated genes, except transcription factors of down-regulated genes, which included 428 transcription factors. In contrast, only three transcription factors were identified in the TSS of down-regulated genes (Figure 1C and Table S4). Among the transcription factors of up-regulated genes, we found 84 transcription factors including well-known age-related transcription factors such as hypoxia-inducible factor 1 alpha (*HIF1α*) and *NFKB2*. Additionally, peroxisome proliferator-activated receptor delta (*PPARδ*) was identified as a transcription factor of down-regulated genes.

**Table 1:** Genes in pathways significantly changed by the aging process

Condition	Function category		Gene members
Up-regulated Genes	Cytokine	CD molecules	CD101, CD163, CD163L1, CD19, CD2, CD22, CD226, CD247, CD300E, CD37, CD38, CD3D, CD3E, CD3G, CD4, CD40LG, CD44, CD5, CD53, CD68, CD69, CD7, CD79B, CD80, CD8B
		Interleukin	IL10RA, IL11, IL18R1, IL18RAP, **IL19**, **IL1R2**, IL1RL1, IL1RN, IL23R, IL24, IL2RA, IL6, IL6R, IL9R
		Interferon	IFI204, IFI27L2B, IFIT3
		Tumor necrosis factor	TNFRSF12A, TNFRSF13B, TNFRSF13C, TNFRSF1B, TNFRSF8, **TNFSF11**, **TNFSF8**
		Chemokine	CCL12, CCL19, CCL2, CCL20, **CCL21**, CCL22, CCL6, CCL7, CCR1, CCR3, CCR5, CCR6, CCR7, **CXCL2**, **CXCL5**, CXCR2, CXCR3, CXCR4, XCR1
	Immune response	B-cell	BCL11A, BCL2A1D, BTK, BTLA, CR2, DAPP1, FCGR2B, PRKCB, RAC2
		T-cell	CTLA4, **FYB**, ICOS, LCK, LCP2, PDCD1, PIK3R5, PTPRC, ZAP70
	Cell adhesion		CLDN14, CLDN4, CTLA4, ITGA4, ITGAL, ITGAM, NCAM1, PDCD1, PTPRC, PVR, SELL, SIGLEC1
	Focal adhesion		BIRC3, COL11A1, COL1A1, COL3A1, COL5A2, COL6A1, FLNA, FLNC, LAMB3, LAMC2, PARVG, PIK3R5, PRKCB, RAC2, SHC2, TNN
	Arachidonic acid metabolism		**ALOX15B**, **ALOX5**, **GPX2**, **PTGIS**, PLA2G2A, PLA2G2D
Down-regulated Genes	Amino acid metabolism		**AFMID**, **ALDH1B1**, **CNDP1**, **EHHADH**, **GRHPR**, **MDH2**
	Fatty acid metabolism		ACAA1B, **ALDH1B1**, EHHADH, CYP4A3
	Circadian rhythm		ARNTL, NPAS2
	Renin angiotensin systems		AGT, REN
	Steroid biosynthesis		DHCR24, DHCR7
	Drug metabolism cytochrome P450		GSTA2, **GSTP1**, **UGT2B15**

We performed a modified gene set enrichment test (see Methods) to classify the 84 transcription factors into their appropriate pathways. Interestingly, many transcription factors of up-regulated genes were involved in cancer or inflammation pathways; pathway in cancer (*P* = 9.0 × 10^−5^), JAK-STAT signaling pathway (*P* = 4.1 × 10^−5^), chemokine pathway (*P* = 7.1 × 10^−5^), and Toll-like receptor (TLR) signaling pathway (*P* = 5.2 × 10^−3^). Moreover, we found that *EGR1* (2.4-fold) and *FOSL1* (4.2-fold), which are involved in both HRAS-related oncogenic pathways [22] and inflammatory responses by LPS [23], were up-regulated with aging.

### Up-regulation of Toll-like receptor signaling leading to inflammation

**Figure 3:**
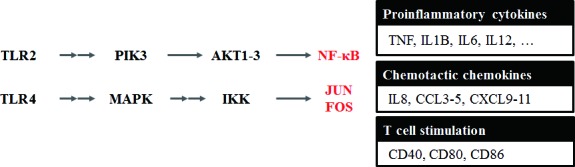
Toll-like receptor signaling pathway changed during aging Based on the gene set enrichment result of up-regulated genes during aging, we analyzed the integrated expression of genes and pathways. We found that the Toll-like receptors (TLRs) family and TLR signaling-related genes were up-regulated during aging. Up-regulation of *NF-κB* and *FOS/JUN* through *TLR2* (2.3-fold) and *TLR4* (2.1-fold) was observed for up-regulation of inflammation-related genes that function in the pro-inflammation effect (*IL-1β*, *IL-6*, *IL-12*, and *TNFα*) and chemotactic genes (*CCL3* and *CD5*), as well as T-cell stimulation related genes (*CD40*, *CD80*, *CD86*, *CXCL9*, *CXCL10*, and *CXCL11*). These data support that pro-inflammatory genes, cytokines, and chemokines were overexpressed through the TLR signaling pathway, leading to chronic inflammation in aged rats. We describe the expression concentration of genes related to TLR signaling pathway in Figure S1 and Table S5.

Based on gene set enrichment results of transcription factors, we analyzed the expression of genes and pathways. We found that TLR family and TLR signaling-related genes were up-regulated during aging (Figure 3 and Figure S1). TLRs are well-known membrane proteins that play a key role in the innate immune system, including the processes of inflammation and antiviral immune responses.

In the TLR signaling pathway, *TLR1-9* proteins are up-regulated during aging, although only *TLR7* (3.5-fold) and *TLR8* (3.2-fold) showed statistically significant p-values, which were calculated by the Cuffdiff program (see Methods). Up-regulation of *NF-κB* and *FOS/JUN* through *TLR2* (2.3-fold) and *TLR4* (2.1-fold) induced up-regulation of inflammation-related genes, which function in the pro-inflammation effect (*IL-1β, IL-6, IL-12*, and *TNF-α*), chemotactic genes (*CCL3* and *CD5*), and T-cell stimulation-related genes (*CD40, CD80, CD86, CXCL9, CXCL10*, and *CXCL11*). These data support that pro-inflammation genes, cytokines, and chemokines are overexpressed through TLRs in aged rats, leading to chronic inflammation. We describe the expression of genes related to the TLR signaling pathways in Table S5.

### Detection of altered arachidonic acid metabolism related to inflammation

Arachidonic acid metabolism has important roles in physiological and pathological processes, including host defense [24], renal disease [25], inflammation [26], and cancer [27]. Arachidonic acid is metabolized to produce eicosanoids (prostaglandins (PG), leukotrienes (LT), prostacyclins) as inflammatory mediators.

We observed that the expression levels of enzymes related to prostaglandin, leukotriene, and thromboxane were significantly changed during aging (Figure S2). Expression of *PGH2* synthase 1 (*PTGS1*, also known as *COX-1*), which plays a role in the synthesis of *PGH2*, leading to improper inflammation states and hypertension during aging, was up-regulated in aged rats by 1.7-fold. Additionally, *TBXAS1*, which is involved in the synthesis of thromboxane A2 (*TXA2*), was up-regulated by 2.1-fold in the aged animals. *TXA2* is mainly produced in the platelets and elicits diverse physiological and pathophysiological reactions, including platelet aggregation and vascular smooth muscle contraction [28].

The expression of enzymes related to the biosynthesis of the leukotriene family of proteins was also altered in the aged animals. 5-Lipoxygenase (*ALOX5,* known as 5-LO), which plays a role in the synthesis of leukotriene A4 (*LTA4*), was up-regulated by more than 6.3-fold with age. In contrast, gamma glutamyl transferase 1 (*GGT1*) and dipeptidase 1 (*DPEP1*) genes, which are involved in the synthesis of leukotriene E4, were down-regulated by 2.0-fold and 1.3-fold respectively. We described the expression of genes related to arachidonic acid metabolism in Table S5.

### Down-regulation of PPARs signaling in kidney

Our analysis detected a change in PPARs and target gene expression. PPARs are important transcription factors to the regulation of several genes involved in lipid metabolism, and they become dis-regulated during aging. In this study, we found that lipoprotein lipase (*LPL*) was increased by 1.5-fold, while *PPARα, PPARδ*, carnitine palmitoyltransferase 1 (*CPT1*), and β-oxidation related genes were decreased with aging by 1.5-, 1.9-, and 1.4- fold, respectively, compared to young kidneys.

*LPL* catalyzes the hydrolysis of the triacylglycerol component of circulating chylomicrons and very low-density lipoproteins, thereby providing non-esterified fatty acids and 2-monoacylglycerol for tissue utilization [29]. Increased renal *LPL* suggests the possibility of lipid accumulation and lipotoxicity in the kidney with aging. This may be an important mechanism in the development of renal injury associated with metabolic syndrome. *CPT* is located in the mitochondrial membranes, and functions to translocate free fatty acids into the mitochondria. Therefore, through increasing *LPL* and decreasing mitochondrial β-oxidation resulting from decreased *PPARα* levels, increased uptake of fatty acids into the kidneys may occur during aging. Our data support that altered lipid metabolism caused by an imbalance between lipogenesis and lipolysis induces subsequent renal lipid accumulation and renal injury [30]. We described the expression concentration of genes related to PPAR signaling in Table S5.

### New gene candidates identified by DEGs

**Figure 4:**
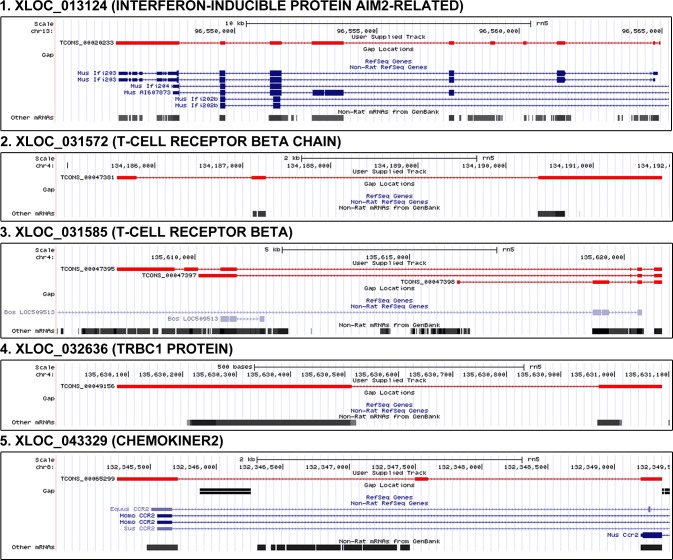
Genomic position and structure of novel genes related to inflammation From 322 novel gene candidates that had at least one homologous gene in the NR database, we found that only 32 contained functional domains. Among the 32 genes, five possessed inflammation-related domains such as the T-cell receptor domain, chemokine domain, and AIM2-related domain. All the gene candidates had significant *P* values (*P* = 1.4 × 10^−5^, Fisher's exact test) in the frequency of inflammation-related genes. We found that XLOC_013124 (5.9-fold increase), which is assigned as an AIM2-realted domain, was similar to the exons of the mouse *AIM2* gene. However, XLOC_013124 showed variation in the number of exons compared to the mouse *AIM2* gene based on our findings. XLOC_031572, XLOC_031585, and XLOC_032636 have T-cell receptor related domains. XLOC_043329 (8.9-fold increase) has a domain of chemokiner2 which is C-C chemokine receptor type 2 signature. In the figure, red color means novel gene.

cDNA microarray technologies cannot detect novel genes on the genomic scale. Because the discovery of new genes is important in aging research, we utilized RNA-Seq data and analyses to identify novel genes that were significantly changed with age. To predict the function of novel genes, we carefully analyzed their sequences using the NCBI NT and domain database after removing noncoding RNA. From 322 novel gene candidates that had at least one homologous gene in the NR database, we found that only 32 contained functional domains. The genes were differentially expressed by more than 2-fold between young and old rats with age. Among the 32 genes, five possessed inflammation-related domains such as the T-cell receptor domain, chemokine domain, and AIM2-related domain (Figure 4). All the gene candidates had significant *P* values (*P* = 1.4 × 10^−5^, Fisher's exact test) in the frequency of inflammation-related genes. *AIM2* is a main component of the inflammasome, which is responsible for activating inflammatory processes [31]. We found that XLOC_013124 (5.9-fold increase), which is assigned as an AIM2-realted domain, was similar to the exons of the mouse *AIM2* gene. However, XLOC_013124 showed variation in the number of exons compared to the mouse *AIM2* gene based on our findings (Figure 4).

### Identification of alternative splicing events during the aging process

Using the Cufflink program, we identified 31,506 known transcripts and 18,119 novel isoforms of known transcripts that occur during aging. This result is similar to the number of novel isoforms previously reported during cell differentiation in mice [19]. To compare the transcripts produced by young and old rats, we selected 107 genes that underwent alternative splicing events and were differentially changed by 2-fold or more.

Among the genes with alternative splicing events, seven genes were specifically related to inflammation (6.5%). Genes related to inflammation included *CCL20, CCR1, CD44, FGG, CXCR3, IL1RN, FCGR2*, and *NFKBIZ*, which were classified as ‘inflammation response’ genes according to GO categories (Table 2). To evaluate the effect of inflammation on alternative splicing events, we tested the statistical significance of inflammation-related genes that had alternative splicing events. In alternative splicing events, the frequency of inflammation-related genes was significant (*P* = 3.3 × 10^−4^, Fisher's exact test), which was similar to known and novel genes.

**Table 2:** Inflammation-related genes with alternative splicing events

Symbol	ref Young	ref Old	FC	alt Young	alt Old	FC	Description
CCL20	0.45	3.80	8.41	0.07	1.01	14.99	C-C motif chemokine 20 precursor
CD44	0.36	1.08	3.00	3.81	17.71	4.65	CD44 antigen precursor
CXCR3	0.50	2.44	4.84	0.70	1.73	2.46	C-X-C chemokine receptor type 3
FCGR2B	0.58	3.55	6.08	0.21	1.35	6.55	low affinity immunoglobulin gamma Fc region receptor II-b precursor
FGG	0.05	0.83	15.34	0.14	0.64	4.65	fibrinogen gamma chain precursor
IL1M	0.64	2.69	4.24	0.16	0.05	0.31	interleukin-1 receptor antagonist protein precursor
NFKBIZ	1.55	4.82	3.10	0.57	2.19	3.86	NF-kappa-B inhibitor zeta

### Real-time PCR results of genes changed by aging

**Figure 5:**
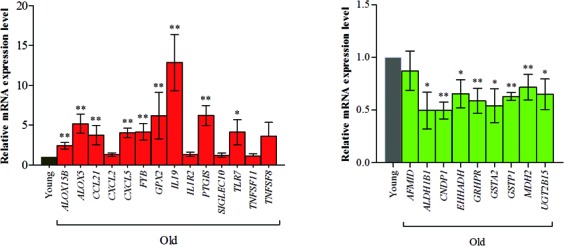
Real-time PCR analysis of genes changed in the aging process We confirmed 23 known genes (14 up-regulated genes and 9 down-regulated genes) using real-time PCR. The genes were selected by expression values and the function for those related to inflammation and metabolism. Among up-regulated genes, arachidonic acid metabolism (*ALOX5, ALOX15B, GPX2*, and *PTGIS*), cell adhesion (*SIGLEC10*), chemokine (*CCL21, CXCL2*, and *CXCL5*), interleukin (*IL19* and *IL1R2*), T-cell signaling (*FYB*), Toll-like receptor (*TLR7*), and tumor necrosis factor (*TNFSF8* and *TNFSF11*) genes were tested for expression levels during aging. In contrast, genes relating to amino acid metabolism (*AFMID, ALDH1B1, CNDP1, GRHPR*, and *MDH2*), drug metabolism (*GSTA2, GSTP1*, and *UGT2B15*), and fatty acid metabolism (*EHHADH*) were tested for down-regulation. We found that the value of fold-change according to RNA-Seq was consistent with the results of real-time PCR analysis during aging (Pearson's correlation = 0.71). Detailed information is described Table S1.

To confirm aged-related gene expression of RNA-Seq data, we performed real-time PCR for 23 known genes (14 up-regulated and nine down-regulated genes). The genes were selected based on their expression values or function related to inflammation or metabolism. Among up-regulated genes, the expression of arachidonic acid metabolism (*ALOX5, ALOX15B, GPX2*, and *PTGIS*), cell adhesion (*SIGLEC10*), chemokine (*CCL21, CXCL2*, and *CXCL5*), interleukin (*IL19* and *IL1R2*), T-cell signaling (*FYB*), Toll-like receptor (*TLR7*), and tumor necrosis factor (*TNFSF8* and *TNFSF11*) were evaluated in young and old animals. In contrast, genes relating to amino acid metabolism (*AFMID, ALDH1B1, CNDP1, GRHPR*, and *MDH2*), drug metabolism (*GSTA2, GSTP1*, and *UGT2B15*), and fatty acid metabolism (*EHHADH*) were tested for down-regulation (Figure 5). Statistical relevance was calculated by Mann-Whitney test. Also, we found that the values for fold-change according to RNA-Seq were consistent with the results of real-time PCR for the old animals. (Pearson's correlation = 0.71, Table S1).

## DISCUSSION

Various cDNA microarray studies have been conducted with limited results to understand aging and they are limited due to the cross-hybridization of probes and measurement of only the relative abundances of transcripts [32]. RNA-Seq is effective in acquiring short but high quality RNA reads [33].

Our RNA-Seq analysis successfully detected the up-regulation of inflammation-related gene candidates that are probably involved in aging. A number of statistically significant (*P* < 0.001) novel genes and alternative splicing events related to inflammation were also detected. In spite of that the results of RNA-Seq should be confirmed by complicated experiments, we showed that inflammation alters gene expression, including expression of previously novel genes and alternative splicing events in the transcriptome of aged rats,

Inflammation was activated through the up-regulation of genes involved in various pathways, such as cytokines, T- and B-cell signaling, TLR signaling, and arachidonic acid metabolism in aged rats although our RNA-Seq analyses are limited and cannot pin-point the causal genes with proper mechanism understanding. This is the limitation of large scale sequencing based analyses.

Arachidonic acid metabolism plays important roles in inflammation and cancer. Changes in eicosanoid-forming enzymes during aging showed interesting results in our experiments. Up-regulation of *COX-1* (1.7-fold) indicated that the key enzyme in *PGH2* synthesis is dysregulated during aging. Increased *TBXAX1* (2.0-fold) may induce synthesis of *TXA2*, which causes changes in platelet shape, aggregation, and secretion, thereby promoting thrombus formation and thrombosis [28]. Several publications report on the overexpression of *TXA2* in aging [34] and several cardiovascular diseases [35]. *ALOX5*, which converts arachidonic acid to *LTA4*, also plays various roles in physiological or pathophysiological conditions [36]. *LTA4* is further changed into *LTB4* by *LTA4* hydroxylase. *LTB4* is related to many inflammatory diseases, such as asthma, inflammatory bowel diseases, atherosclerosis, and nephritis [37]. Despite its importance in inflammation, changes in LT synthase enzymes such as *ALOX5* with aging were relatively negligible. We observed that *ALOX5* gene expression levels were up-regulated in aged kidneys, implying that this up-regulation may aggravate age-related kidney dysfunction.

PPARs are ligand-activated transcription factors belonging to the nuclear receptor superfamily. Although PPAR involvement was thought to be limited to lipid metabolism and glucose homeostasis, recent studies show that PPARs are also important in aging processes and age-related inflammation. *PPARα* has been demonstrated to play an important role in regulating the *CPT* gene. Reduced *PPARα* expression down-regulates *CPT2* gene expression, leading to renal lipid accumulation. Previous studies have shown that age-associated declines in PPARα expression and function occur in a variety of tissues, including the kidney. Similarly, the expression of several genes encoding β-oxidation enzymes, which are regulated by *PPARα*, is also shown to be reduced as a consequence of aging. Our data are in agreement with previously published data obtained with the down-regulation of *PPARα* levels in RNA-Seq data. Reduction of PPARα with aging may be related to the observed aging-associated effects on lipid metabolism and fatty acid oxidation activity in the kidney.

**Figure 6:**
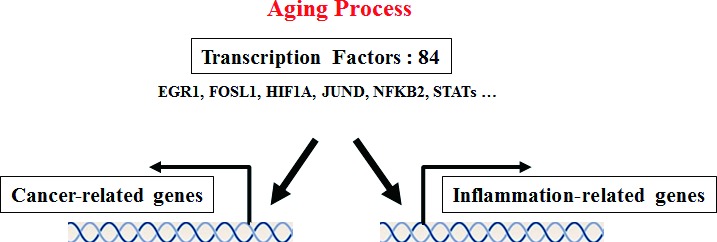
Proposed common transcription factors linking inflammation and cancer in the aging process To identify regulatory molecules of up-regulated genes obtained from the gene set enrichment test, we conducted transcription factor analysis using the TRANSFAC database. We found that 84 transcription factors existed at the transcriptional start site of up-regulated genes. The current transcription factor analysis showed that transcription factors of up-regulated genes can regulate genes related to both inflammation and cancer. The transcription factors included well-known cancer-related factors such as *EGR1, FOSL1, HIF1A, JUND, NFKB2*, and *STATs*. These transcription factors may play major roles to link cancer and inflammatory aging, and they may be target molecules to research the correlation between cancer and aging. Detailed information is described in Table S4.

This current transcription factor analysis showed that transcription factors of up-regulated genes can regulate genes related to both inflammation and cancer. The transcription factors included well-known cancer-related factors such as *EGR1, FOSL1, HIF1A, JUND, NFKB2*, and *STATs*. These results indicate that transcription factors are important links between cancer and the inflammatory aging process, and are good target molecules in identifying the correlation between cancer and aging, as depicted in Figure 6.

In summary, we conducted a large scale RNA-Seq to obtain highly accurate transcriptome data, which is useful for exploring complex pathophysiological phenomena such as aging. The RNA-Seq data and analyses enabled us to identify transcriptome changes, including novel genes and alterative splicing forms altered by aging. We also speculate that RNA-Seq data is useful to detect candidate markers and can be used as diagnostics to provide continuous monitoring of pathology related to aging.

## MATERIALS AND METHODS

### Sample preparation for Illumina Hiseq2000 sequencing

Young (6-month-old) and old (25-month-old) specific pathogen-free male Sprague-Dawley rats were obtained from Samtako, Inc. (Osan, Korea). Rat maintenance procedures for specific pathogen-free status and dietary composition of chow have been previously reported [19, 38]. The cortex of kidney tissues from the six SD rats was removed for RNA extraction. Rats were sacrificed by decapitation and the kidneys were quickly removed and immediately frozen in liquid nitrogen and stored at −80°C. Total RNA was isolated from the cortex of kidney sample using the miRNeasy Mini Kit (Qiagen, Hilden, Germany). An RNA sequencing library was generated using TruSeq RNA sample preparation Kit according to user's instruction manual (Illumina, San Diego, CA, USA). Briefly, mRNA was separated from total RNA using Oligo(dT) beads and chemically fragmented. After double-strand cDNA synthesis of the fragmented mRNA, end-repair, adenylation of 3′-end, and sequencing adapter ligation were performed, followed by DNA purification with magnetic beads and PCR amplification. Finally, the amplified library was purified, quantified, and then applied for template preparation. The HiSeq2000 platform was utilized to generate 99-bp paired-end sequencing reads (Illumina).

### Genome mapping and identification of paired-end sequences

All 99-bp paired-end sequence reads were mapped to the rat genome (NCBI build 5) using TopHat version 2.0.4. These mapped reads were merged for each condition (young vs old), and transfrags were assembled using Cufflink version 2.0.1. These merged transfrags were quantified for each condition using the Cuffdiff program. Finally, we identified differentially expressed genes, novel alternative splicing isoforms, and novel expression of transcript candidates.

### Gene set enrichment test

To characterize the biological pathways related to differentially expressed sequences and transcription factors, representative pathways were analyzed in the context of several databases such as KEGG (http://www.genome.ad.jp), BioCarta (http://www.biocarta.com), and Reactome (http://www.reactome.org), as suggested by MsigDB v4.0 [20]. The pathway terminologies are listed in MsigDB. Additionally, we used Fisher's exact test and FDR to examine mapping pathways (filtering options: *P* < 0.01 and FDR < 5). FDR was calculated by q-value package of R program. We developed a modified gene set enrichment test to obtain the *p*-value of the gene set involving transcription factors that existed in the TSS of up-regulated inflammatory gene. Because number of transaction factors in a pathway were smaller than other proteins, we multiplied the gene set of transcription factors (*P_gsea_*(*TF*)) by gene set of total differentially expressed genes ((*P_gsea_*(*DEG_total_*)). *P_TF_* indicates the modified p-value that was calculated using the following equation:

$$P_{\text{TF}}=\sqrt{P_{\text{gsea}}(TF_{\text{up}}-TF_{\text{down}})\times P_{\text{gsea}}(DEG_{\text{total}})}$$

### Gene ontology analysis and functional annotation

To identify inflammation-related genes, we used GO terms, which are part of an international standardized gene functional classification system. The GO annotations of each gene were obtained from gene2go, which is a part of the NCBI Entrez database. We regarded genes with ‘inflammation response’ in the GO term as the inflammation-related genes. To determine the function of novel genes, we aligned our sequences with the nucleotide databases of NT (filtering option: e-value < 0.0001) using BLAST. The gene showing the highest sequence similarity in each BLAST result was chosen to annotate the function of the query gene. From novel genes, which were assigned to the NCBI NT database, we removed non-coding RNA using BLAST alignment tool using Rfam database [39] (filtering option: e-value < 0.0001). To predict whether a functional domain existed in novel genes, we also used InterProScan version 5 with a filtering option of *P* < 1 × 10^−4^ [40].

### Quantitative RT-PCR

Total RNA from the tissues was isolated using a rapid extraction method (TRI-Reagent, Invitrogen, Carlsbad, CA, USA). Real-time PCR was performed on cDNA samples using the iQTM SYBR Green Supermix system (Bio-Rad, Hercules, CA, USA). Primers are described in Table S1. The protocols used are as follows: denaturation (95°C for 2 min), amplification repeated 40 times (95°C for 15 s, 55°C for 30 s, 72°C for 30 s, and acquisition temperature for 15 s) (Bioneer ExicycleTM 96, Daejeon, Korea). Analysis was conducted using the sequence detection software supplied with the instrument. For each sample, the delta delta Threshold Cycle (ddCT) (crossing point) values were calculated as the Ct of the target gene minus the Ct of the GAPDH gene. Gene expression was derived according to the equation 2–ddCt; changes in gene expression are expressed relative to basal levels.

## SUPPLEMENTARY MATERIAL FIGURES AND TABLES













## References

[1] Ames BN, Shigenaga MK, Hagen TM (1993). Oxidants, antioxidants, and the degenerative diseases of aging. Proc Natl Acad Sci U S A.

[2] Chung HY, Kim HJ, Kim KW, Choi JS, Yu BP (2002). Molecular inflammation hypothesis of aging based on the anti-aging mechanism of calorie restriction. Microsc Res Tech.

[3] Chung HY, Lee EK, Choi YJ, Kim JM, Kim DH, Zou Y, Kim CH, Lee J, Kim HS, Kim ND, Jung JH, Yu BP (2011). Molecular inflammation as an underlying mechanism of the aging process and age-related diseases. J Dent Res.

[4] Franceschi C (2007). Inflammaging as a major characteristic of old people: can it be prevented or cured?. Nutr Rev.

[5] Salminen A, Kaarniranta K, Kauppinen A (2012). Inflammaging: disturbed interplay between autophagy and inflammasomes. Aging (Albany NY).

[6] Lavrovsky Y, Chatterjee B, Clark RA, Roy AK (2000). Role of redox-regulated transcription factors in inflammation, aging and age-related diseases. Exp Gerontol.

[7] Vasto S, Candore G, Balistreri CR, Caruso M, Colonna-Romano G, Grimaldi MP, Listi F, Nuzzo D, Lio D, Caruso C (2007). Inflammatory networks in ageing, age-related diseases and longevity. Mech Ageing Dev.

[8] de Magalhaes JP, Curado J, Church GM (2009). Meta-analysis of age-related gene expression profiles identifies common signatures of aging. Bioinformatics.

[9] Lu T, Pan Y, Kao SY, Li C, Kohane I, Chan J, Yankner BA (2004). Gene regulation and DNA damage in the ageing human brain. Nature.

[10] Zahn JM, Poosala S, Owen AB, Ingram DK, Lustig A, Carter A, Weeraratna AT, Taub DD, Gorospe M, Mazan-Mamczarz K, Lakatta EG, Boheler KR, Xu X, Mattson MP, Falco G, Ko MS (2007). AGEMAP: a gene expression database for aging in mice. PLoS Genet.

[11] Cloonan N, Forrest AR, Kolle G, Gardiner BB, Faulkner GJ, Brown MK, Taylor DF, Steptoe AL, Wani S, Bethel G, Robertson AJ, Perkins AC, Bruce SJ, Lee CC, Ranade SS, Peckham HE (2008). Stem cell transcriptome profiling via massive-scale mRNA sequencing. Nat Methods.

[12] Mortazavi A, Williams BA, McCue K, Schaeffer L, Wold B (2008). Mapping and quantifying mammalian transcriptomes by RNA-Seq. Nat Methods.

[13] Hanley MB, Lomas W, Mittar D, Maino V, Park E (2013). Detection of low abundance RNA molecules in individual cells by flow cytometry. PLoS One.

[14] Zhang L, Zhou W, Velculescu VE, Kern SE, Hruban RH, Hamilton SR, Vogelstein B, Kinzler KW (1997). Gene expression profiles in normal and cancer cells. Science.

[15] Inukai S, de Lencastre A, Turner M, Slack F (2012). Novel microRNAs differentially expressed during aging in the mouse brain. PLoS One.

[16] Wood SH, Craig T, Li Y, Merry B, de Magalhaes JP (2013). Whole transcriptome sequencing of the aging rat brain reveals dynamic RNA changes in the dark matter of the genome. Age (Dordr).

[17] Marioni JC, Mason CE, Mane SM, Stephens M, Gilad Y (2008). RNA-seq: an assessment of technical reproducibility and comparison with gene expression arrays. Genome Res.

[18] Trapnell C, Pachter L, Salzberg SL (2009). TopHat: discovering splice junctions with RNA-Seq. Bioinformatics.

[19] Trapnell C, Williams BA, Pertea G, Mortazavi A, Kwan G, van Baren MJ, Salzberg SL, Wold BJ, Pachter L (2010). Transcript assembly and quantification by RNA-Seq reveals unannotated transcripts and isoform switching during cell differentiation. Nat Biotechnol.

[20] Subramanian A, Tamayo P, Mootha VK, Mukherjee S, Ebert BL, Gillette MA, Paulovich A, Pomeroy SL, Golub TR, Lander ES, Mesirov JP (2005). Gene set enrichment analysis: a knowledge-based approach for interpreting genome-wide expression profiles. Proc Natl Acad Sci U S A.

[21] Wingender E, Chen X, Hehl R, Karas H, Liebich I, Matys V, Meinhardt T, Pruss M, Reuter I, Schacherer F (2000). TRANSFAC: an integrated system for gene expression regulation. Nucleic acids research.

[22] Bild AH, Yao G, Chang JT, Wang Q, Potti A, Chasse D, Joshi MB, Harpole D, Lancaster JM, Berchuck A, Olson JA, Marks JR, Dressman HK, West M, Nevins JR (2006). Oncogenic pathway signatures in human cancers as a guide to targeted therapies. Nature.

[23] Seki E, De Minicis S, Osterreicher CH, Kluwe J, Osawa Y, Brenner DA, Schwabe RF (2007). TLR4 enhances TGF-beta signaling and hepatic fibrosis. Nat Med.

[24] Shea JM, Del Poeta M (2006). Lipid signaling in pathogenic fungi. Curr Opin Microbiol.

[25] Camara NO, Martins JO, Landgraf RG, Jancar S (2009). Emerging roles for eicosanoids in renal diseases. Curr Opin Nephrol Hypertens.

[26] Khanapure SP, Garvey DS, Janero DR, Letts LG (2007). Eicosanoids in inflammation: biosynthesis, pharmacology, and therapeutic frontiers. Curr Top Med Chem.

[27] Wang D, Dubois RN (2010). Eicosanoids and cancer. Nat Rev Cancer.

[28] Nakahata N (2008). Thromboxane A2: physiology/pathophysiology, cellular signal transduction and pharmacology. Pharmacol Ther.

[29] Mead JR, Irvine SA, Ramji DP (2002). Lipoprotein lipase: structure, function, regulation, and role in disease. J Mol Med (Berl).

[30] Kume S, Uzu T, Araki S, Sugimoto T, Isshiki K, Chin-Kanasaki M, Sakaguchi M, Kubota N, Terauchi Y, Kadowaki T, Haneda M, Kashiwagi A, Koya D (2007). Role of altered renal lipid metabolism in the development of renal injury induced by a high-fat diet. J Am Soc Nephrol.

[31] Fernandes-Alnemri T, Yu JW, Datta P, Wu J, Alnemri ES (2009). AIM2 activates the inflammasome and cell death in response to cytoplasmic DNA. Nature.

[32] Irizarry RA, Warren D, Spencer F, Kim IF, Biswal S, Frank BC, Gabrielson E, Garcia JG, Geoghegan J, Germino G, Griffin C, Hilmer SC, Hoffman E, Jedlicka AE, Kawasaki E, Martinez-Murillo F (2005). Multiple-laboratory comparison of microarray platforms. Nat Methods.

[33] Baginsky S, Hennig L, Zimmermann P, Gruissem W (2010). Gene expression analysis, proteomics, and network discovery. Plant Physiol.

[34] Chung HY, Kim HJ, Shim KH, Kim KW (1999). Dietary modulation of prostanoid synthesis in the aging process: role of cyclooxygenase-2. Mech Ageing Dev.

[35] Michelson AD (2010). Antiplatelet therapies for the treatment of cardiovascular disease. Nat Rev Drug Discov.

[36] Harizi H, Corcuff JB, Gualde N (2008). Arachidonic-acid-derived eicosanoids: roles in biology and immunopathology. Trends Mol Med.

[37] Wymann MP, Schneiter R (2008). Lipid signalling in disease. Nat Rev Mol Cell Biol.

[38] Iwasaki K, Gleiser CA, Masoro EJ, McMahan CA, Seo EJ, Yu BP (1988). Influence of the restriction of individual dietary components on longevity and age-related disease of Fischer rats: the fat component and the mineral component. J Gerontol.

[39] Nawrocki EP, Burge SW, Bateman A, Daub J, Eberhardt RY, Eddy SR, Floden EW, Gardner PP, Jones TA, Tate J, Finn RD (2015). Rfam 12. 0: updates to the RNA families database. Nucleic acids research.

[40] Quevillon E, Silventoinen V, Pillai S, Harte N, Mulder N, Apweiler R, Lopez R (2005). InterProScan: protein domains identifier. Nucleic acids research.

