# Supplementary material for: RNA-Seq analysis reveals new evidence for inflammation-related changes in aged kidney
Source: Oncotarget. 2016 May 3;7(21):30037–48. doi: 10.18632/oncotarget.9152 (PMC5058662; doi:10.18632/oncotarget.9152)
Supplement: Supplementary file 1 [file oncotarget-07-30037-s001.pdf]

# RNA-Seq analysis reveals new evidence for the inflammation-related changes in aged kidney

## Supplementary Material

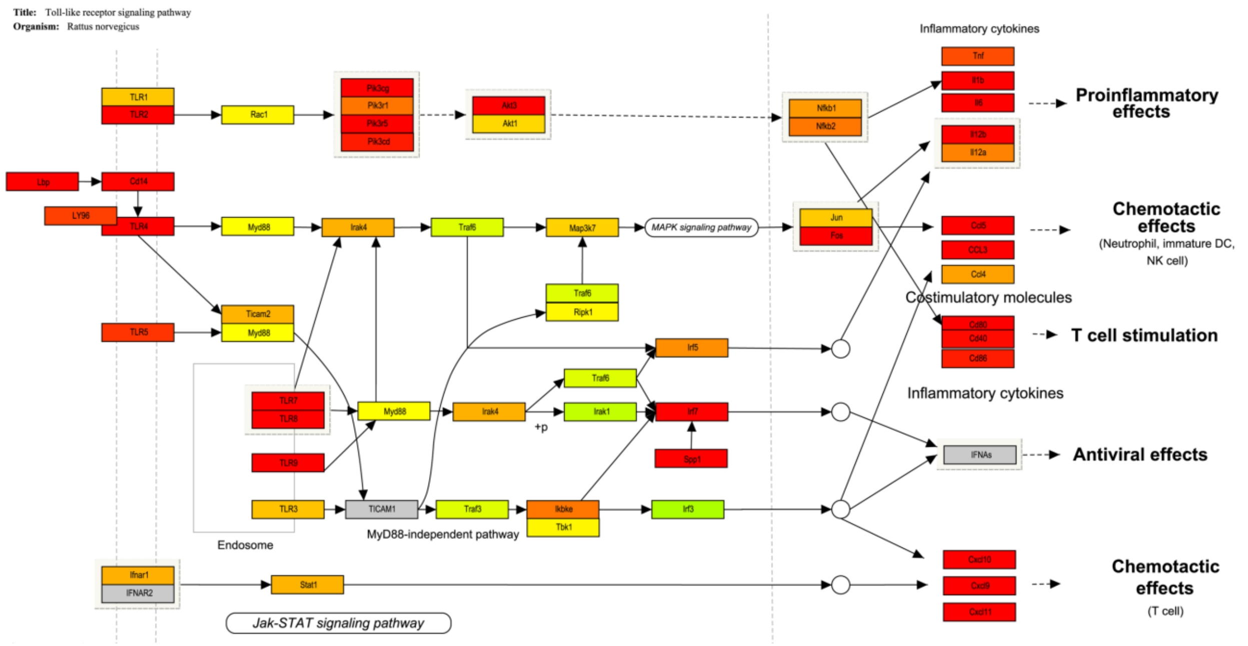


Fig. S1 Toll-like receptor signaling pathway

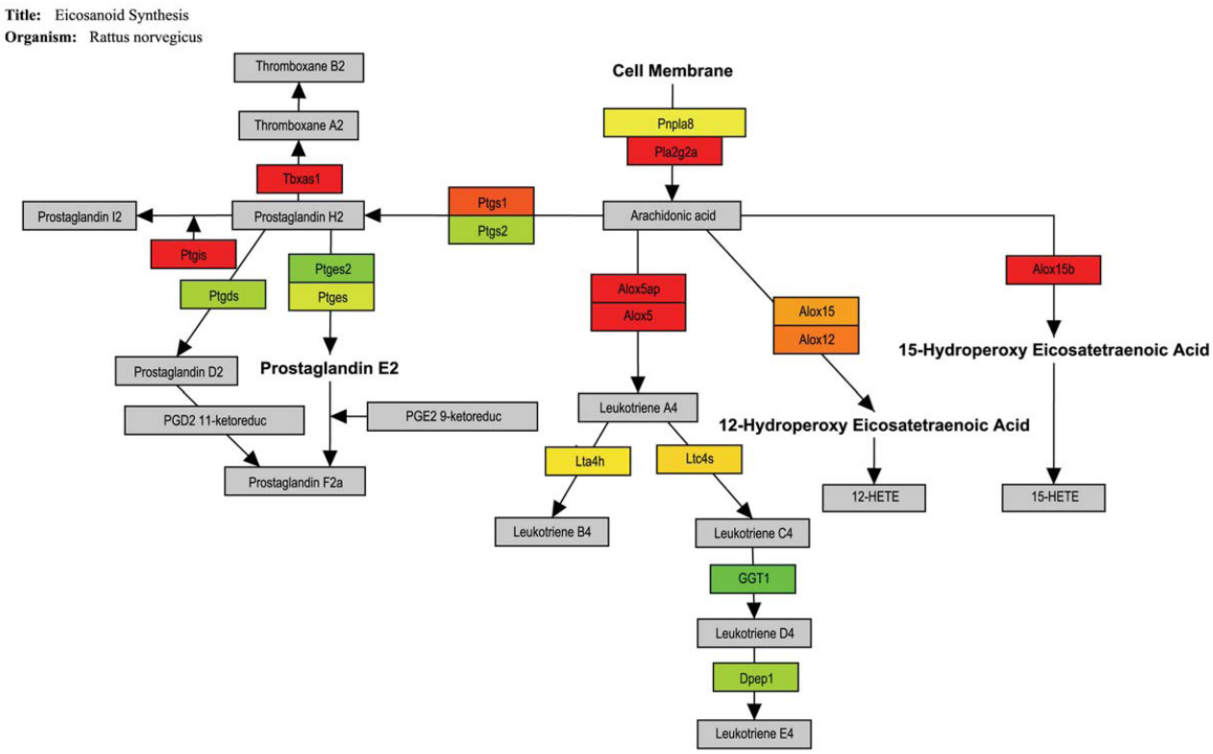


Fig. S2 Arachidonic acid metabolism

Table S1. Primer sequences used for real-time PCR

Table S2. Significantly changed genes during aging process

Table S3. Results of gene set enrichment test using differentially expressed genes

Table S4. Transcription factors of differentially expressed genes during aging process

Table S5. Genes including TLR signaling, arachidonic acid metabolism, and PPAR related pathway
